# Societal Attitudes Towards Transgender Individuals: A Multicentric Survey Across Urban, Rural and Cosmopolitan Cities in India

**DOI:** 10.1192/bjo.2025.10194

**Published:** 2025-06-20

**Authors:** Arokia Antonysamy, Amutha Govindaraju, Helena Selvakodi, Shanmugapriya Samiyappan

**Affiliations:** ^1^Cygnet Health Care, London, United Kingdom; ^2^Madurai Medical College Hospitals, Madurai, India

## Abstract

**Aims:** The aim of this study is to investigate public attitudes towards transgender individuals, seeking to understand the relationship between socio-demographic factors, gender beliefs and approach towards this minority group. The research acknowledges the widespread stigma faced by transgender people due to the incongruence between their gender identity and sex assigned at birth, which negatively impacts their access to resources and overall well-being.

**Methods:** This is a cross-sectional survey conducted across rural, suburban and cosmopolitan cities in India. A total of 500 participants were randomly selected using multistage sampling. Inclusion criteria for participants included the age range 18–60 years, no history of mental illness, and having no known family members with transgender characteristics.

This specific demographic targeting aimed to isolate general public perception, excluding potentially biased viewpoints from those with direct personal connections to transgender individuals or pre-existing mental health conditions.

**Results:** The study revealed a predominantly young adult sample, with 52.6% of respondents falling between the ages of 26 and 40. The sample was fairly distributed across both genders, male (56%), females (44%). Majority were married (62.4%), educated at graduate level (61%) and employed. This socio-demographic breakdown provides context for interpreting the attitudinal data.

Key findings indicated a concerning trend: younger, unmarried males with higher education demonstrated more negative attitudes towards transgender individuals. This finding contradicts some expectations, as higher education is often associated with more liberal and inclusive viewpoints. Furthermore, the study found that highly educated individuals, in general, held more negative attitudes, while married individuals tended to express more positive attitudes. Interestingly, there was no significant difference in attitudes towards transgender men compared with transgender women.

**Conclusion:** The study concludes by emphasizing the need for targeted educational interventions, particularly aimed at younger, unmarried, highly educated males. The authors argue that these interventions are crucial for promoting cultural competence and ensuring that transgender individuals are afforded their due rights. By focusing on education and awareness, the study suggests a potential pathway for mitigating the negative attitudes identified and fostering a more inclusive and accepting society for transgender individuals. The study highlights the importance of understanding the complex interplay between socio-demographic factors and attitudes towards marginalized groups to develop effective strategies for social change.

